# Second District Dental Society of New York

**Published:** 1872-05

**Authors:** J. C. Wyman

**Affiliations:** Sect'y.


### ARTICLE IV.

### *Second District Dental Society of New York.*

The annual meeting of this Society was held in Brooklyn on Tuesday, April 9th, 1872.

After some miscellaneous business had been transacted, the election of officers was held with the following result:

*President.*—Dr. O. E. Hill, of Brooklyn.

*Vice President.*—Dr. T. C. Royce, of Middletown.

*Treasurer.*—Dr. H. G. Merrick, of Brooklyn.

*Recording Secretary.*—Dr. M. E. Elmendorf, of Brooklyn.

*Corresponding Secretary.*—Dr. F. W. Dolbean, of Brooklyn.

*Librarian.*—Dr. G. A. Mills, of Brooklyn.

Delegates to State Society.—Dr. W. B. Hurd, and Dr. W. S. Elliott.

In the course of the proceedings a call was made upon the committee appointed by the convention of dentists in

1871, for information in regard to the rubber suits. This call or request being approved by the Society, Dr. Hill and Dr. Northrup, who were present, gave a detailed statement of the entire proceedings of the committee, since their appointment, of the difficulties they had encountered, of the presentation and argument of the case in the Supreme Court, and of the hope of success which the committee entertained.

The annual dinner was given in the evening, at which many distinguished visitors were present, and among them Rev. Dr. Moore, of Cincinnati, Ohio.

Upon reconvening, the President elect, was formally inducted into the chair, and the remaining business of the meeting transacted.

The following resolution was adopted with the direction that it be offered to the severals dental journal for publication.

*Resolved*, That the thanks of this Society are hereby tendered to the committee appointed to the convention of dentists, held in New York city, in December last, to prosecute the interests of the profession in the matter of the Rubber Patents, and also to the counsel employed by said committee, for the very thorough and able manner in which those interests were vindicated before the Supreme Court of the United States, and let the result be what it may, we hereby record our approval of their action, our thanks for their unwearied efforts, and our sympathy with them in all the difficulties and under all the adverse circumstances which they have met in the execution of their trust.

The meeting then adjourned.

M. E. Elmendorf, Sec'y.

At a meeting of the Brooklyn Dental Society, Monday evening, April 15th, the following action was had:

*Resolved*, That this Society affirm and endorse the resolution of thanks and approbation passed at the annual meeting of the Second District Dental Society, April 9th, as to the action of the committee on the patent suit and their counsel.

Jno. C Wyman, Sec'y.

